# Hidden players of COVID-19: the evolving roles of SARS-CoV-2 accessory proteins

**DOI:** 10.3389/fimmu.2025.1726698

**Published:** 2025-11-28

**Authors:** Miguel Padilla-Blanco, Tránsito García-García, Juozas Grigas, Blanca D. López-Ayllón, Juan J. Garrido, María A. Oliva, María Montoya

**Affiliations:** 1 Viral immunology laboratory, Biomedicine Department, BICS unit. Margarita Salas Centre for Biological Research (CIB-CSIC), Madrid, Spain; 2 Unit for the Development of Biological, Immunological and Chemical Drugs (BICS). Centro de Investigaciones Biológicas Margarita Salas (CSIC), Madrid, Spain; 3 Department of Genetics, Immunogenomics and Molecular Pathogenesis Group, UIC Zoonoses and Emergent Diseases ENZOEM, University of Córdoba, Córdoba, Spain; 4 Maimónides Biomedical Research Institute of Córdoba (IMIBIC), GA-14 Research Group, Córdoba, Spain; 5 Department of Anatomy and Physiology, Lithuanian University of Health Sciences, Kaunas, Lithuania; 6 Institute of Microbiology and Virology, Lithuanian University of Health Sciences, Kaunas, Lithuania; 7 Department of Immunology, Institute of Biotechnology, Life Sciences Centre, Vilnius University, Vilnius, Lithuania; 8 Biological Defense Area, CBRN Defense Department, General Subdirectorate for Terrestrial Systems, National Institute for Aerospace Technology (INTA), Madrid, Spain

**Keywords:** SARS-CoV-2, COVID-19, accessory proteins, viral pathobiology, omicron lineages

## Abstract

SARS-CoV-2 accessory proteins (APs), particularly ORF3a and ORF9b, have emerged as key modulators of host-pathogen interaction and potential contributors to long COVID. Of the 13 predicted APs, only nine are expressed during infection – termed *Infection-related APs* – while the remaining are classified as *Putative APs*. Despite this distinction, extensive gene overlap among APs underscores the remarkable adaptability of SARS-CoV-2 viral genome. This review delves into the diverse roles of the original Wuhan APs and their Omicron counterparts in shaping host immunity, with an emphasis on their ability to suppress type I interferon (IFN-I) signalling, modulate cellular metabolism, and trigger inflammatory/apoptotic pathways. By integrating immunopathological insights with evolutionary dynamics and structural perspectives, this review provides a comprehensive understanding of the mechanism underlying Omicron’s reduced pathogenicity and highlights promising, yet unexplored, therapeutic targets within the SARS-CoV-2 accessory proteome.

## 1 Introduction

Given its central role in viral infectivity and immunogenicity, early SARS-CoV-2 research primarily focused on spike (S) protein, which mediates binding to the host cell receptor angiotensin-converting enzyme 2 (ACE2) (1). However, SARS-CoV-2 viral genome encodes several additional proteins that initially received less attention but have since attracted growing interest. Among these are accessory proteins (APs), key modulators of diverse cellular processes, including inhibition of type I interferon response (IFN-I) (2), induction of proinflammatory cytokine production and apoptosis (8), and reprogramming of cellular metabolism (3, 4). A summary of current knowledge regarding SARS-CoV-2 APs is provided in **Supplementary Table 1**.

APs are present in most coronavirus genomes, including the highly pathogenic SARS-CoV and MERS-CoV, where they play similar roles in modulating the host response (5). Moreover, distantly related viruses such as HIV-1 and influenza A virus also encode APs that interfere with host defence mechanisms, including IFN-I signalling and inflammatory responses (6–8). This widespread occurrence of APs across diverse viral families highlights their evolutionary significance and suggest that examining the evolution of SARS-CoV-2 APs could uncover conserved strategies of immune modulation and viral adaptation.

**Figure 1:**
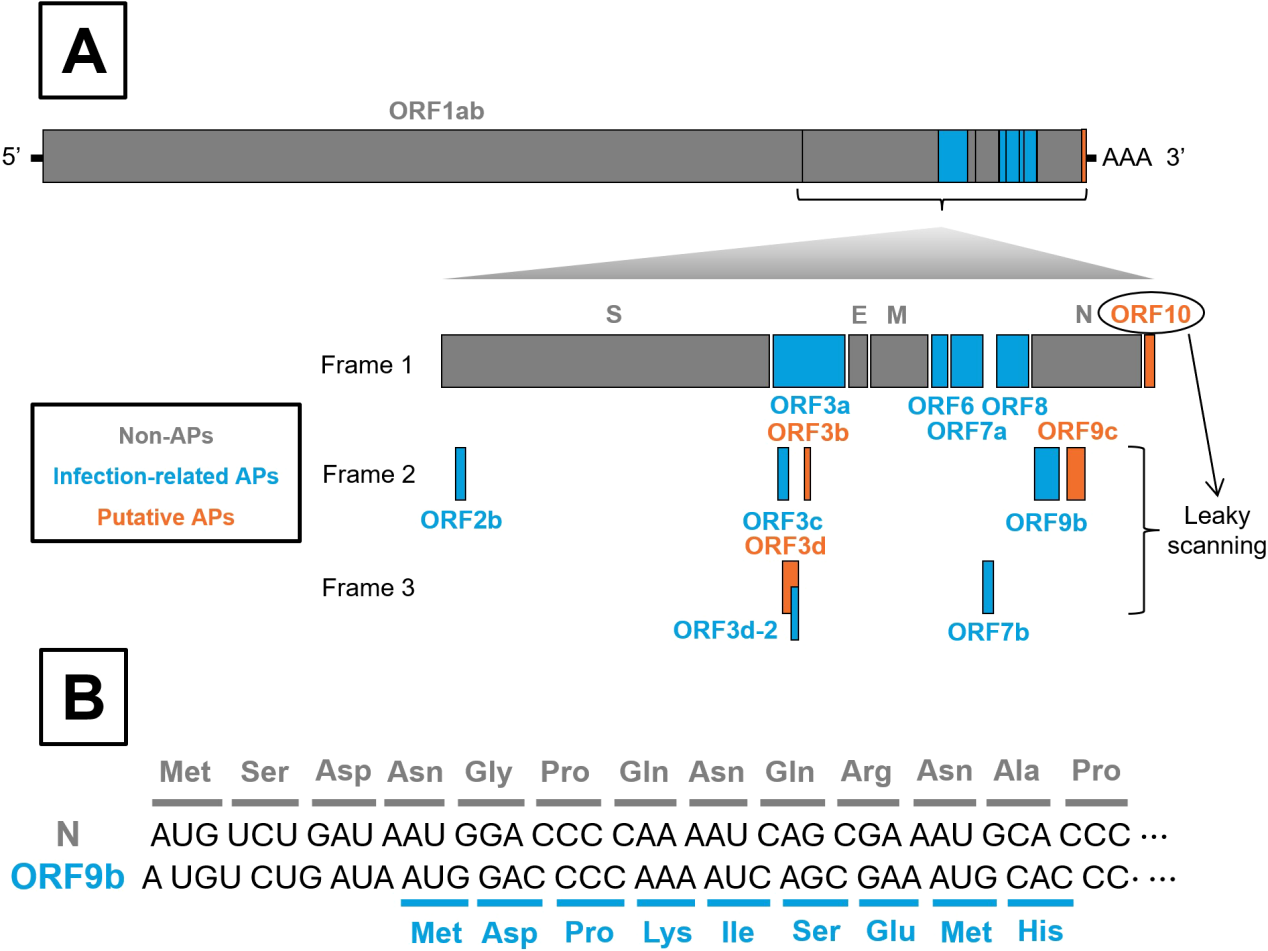
Schematic organization of SARS-CoV-2 genome and leaky scanning process. **(A)** All SARS-CoV-2 genes are shown, with particular emphasis on accessory proteins (APs), whether or not they are synthesized as a result of the leaky scanning process. Non-APs, Infection-related APs and Putative APs are colored in grey, blue and orange, respectively. **(B)** Illustration of the leaky scanning process, exemplified by ORF9b translation.

In SARS-CoV-2, APs are encoded in the last third of the viral genome, interspaced with the structural protein coding genes (*spike* [*S*], *envelope* [*E*], *matrix* [*M*] and *nucleocapsid* [*N*]). They are located downstream of *ORF1ab*, which encodes the viral replication complex (**Figure 1A**). At least nine APs (ORF2b, ORF3a, ORF3c, ORF3d-2, ORF6, ORF7a, ORF7b, ORF8, and ORF9b) have been experimentally confirmed to be expressed during infection (9). In addition, four other proteins (ORF3b, ORF3d, ORF9c and ORF10) have been predicted bioinformatically but remain undetected in infected cells (10–12). Interestingly, exogenous overexpression of some of these predicted proteins can significantly disrupt cellular processes (3, 10, 13). Accordingly, SARS-CoV-2 APs can be classified into two groups: *Infection-related APs* and *Putative APs*, respectively (**Figure 1A**).

Regardless of such classification, several AP-encoding genes overlap with genes encoding larger proteins. For instance, *ORF2b* overlaps with *S*; *ORF9b* and *ORF9c* overlap with *N*; and *ORF3b*, *ORF3c*, *ORF3d*, and *ORF3d-2* overlap with *ORF3a*, which encodes the largest AP. Moreover, *ORF3c* and *ORF3d* partially overlap with each other (**Figure 1A**) (10, 14). These overlapping regions can be translated through a process known as leaky scanning (**Figure 1B**), which occurs when ribosome bypass the first AUG start codon as they scan the RNA in the 5’ to 3’ direction. Translation then initiates at a downstream AUG codon, potentially shifting the open reading frame and producing alternative proteins. This mechanism can reduce expression of the primary gene in favour of overlapping genes (15). Notably, overlapping genes appear to confer an evolutionary advantage to SARS-CoV-2 infection, as they have remained largely conserved throughout viral evolution, and premature stop codon within these regions are rare (16).

In this context, this review summarizes the roles of SARS-CoV-2 APs in host-pathogen interactions, identifying key gaps in current knowledge and discussing future research directions. To achieve this, we integrated for the first time evolutionary dynamics, structural perspectives and immunopathological insights, providing a comprehensive overview of SARS-CoV-2´s APs. This integrative approach is crucial to understand the reduced pathogenicity of Omicron, the currently circulating variant (17). Both infection-related and putative APs are analyzed under this framework. Notice, however, that the roles of putative APs have only been investigated through overexpressing their predicted protein sequences in cultured cell lines, as these proteins have not yet been detected during natural SARS-CoV-2 infection. The discussion that follows examines the function and impact of each ORF in numerical order, regardless of their relative prominence in each section.

## 2 Modulation of host immune responses

### 2.1 Suppression of Type I interferon signalling

**Figure 2:**
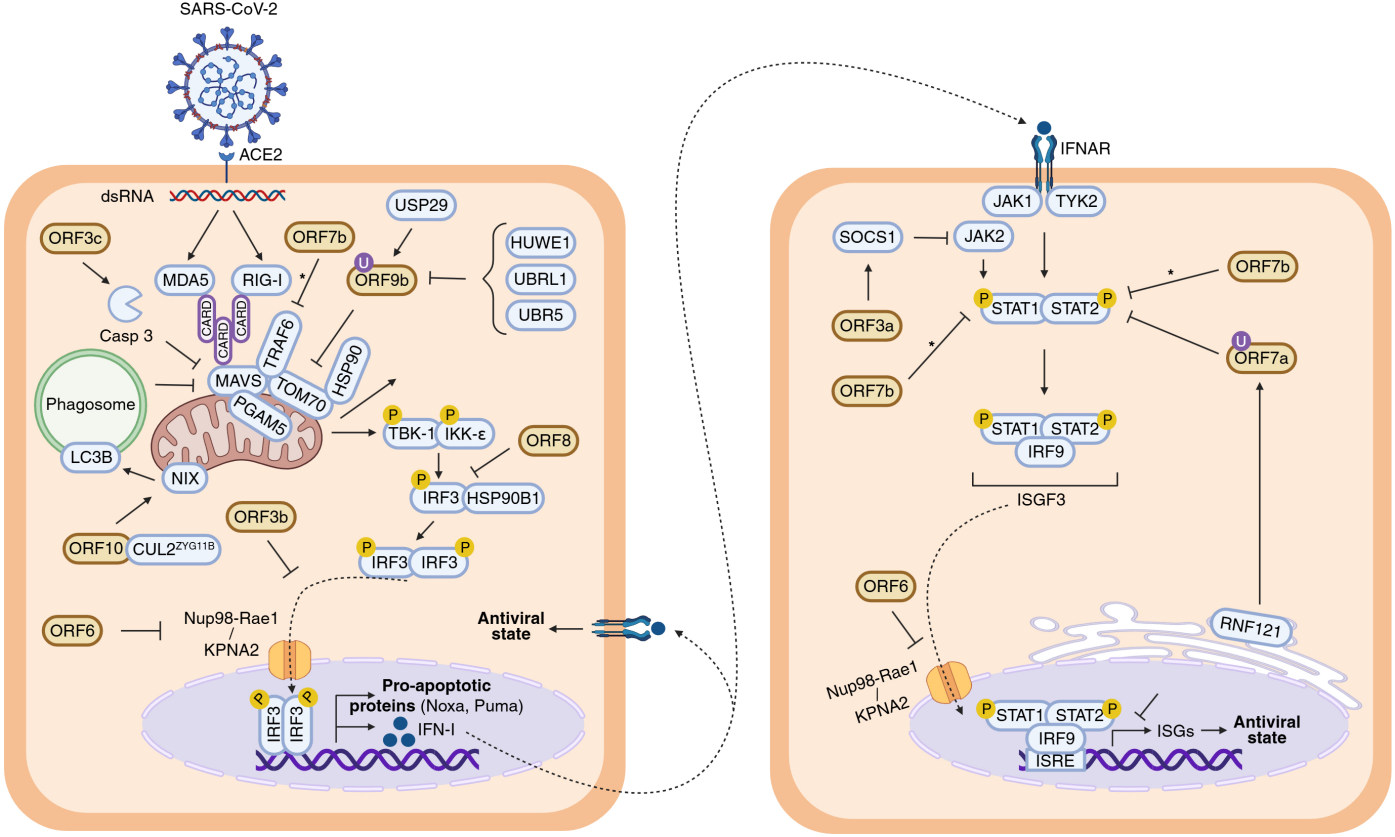
Inhibition of IFN-I response by SARS-CoV-2 APs. Schematic diagram illustrating the roles of SARS-CoV-2 APs in inhibiting the IFN-I response from viral entry and internalization of its genetic material onward. The left cell represents the signalling cascade leading to IFN-I production, whereas the right cell depicts the downstream signalling pathway leading to the expression of ISGs. The antiviral state is indicated in both cells to emphasize the autocrine (left) and paracrine (right) nature of IFN-I response. Inconclusive results are indicated with an asterisk (*). Figure created with Biorender.com.

Cells activate the IFN-I response against viral pathogens (**Figure 2**), triggered by viral double-stranded RNA (dsRNA) or replication intermediates (18, 19). This response begins with the recognition of viral RNA by two cytosolic pattern recognition receptors (PRRs), RIG-I and MDA5, which subsequently expose their CARD domains to interact with the mitochondrial adaptor MAVS. MAVS then recruits downstream signalling proteins that phosphorylate the TBK1 and IKKϵ kinases. In turn, these kinases phosphorylate IRF3, promoting its homodimerization, nuclear translocation and transcription of type I interferons (IFN-I). Secreted IFN-I binds to IFNAR receptors, activating JAK1 and TYK2 kinases, which phosphorylate STAT1 and STAT2. These form the ISGF3 complex with IRF9, which translocate to the nucleus, bind interferon-sensitive response elements (ISREs) and, induce transcription of hundreds of interferon-stimulated genes (ISGs). ISG-coded proteins inhibit viral genome replication and protein synthesis, with examples including MX1, PKR, OAS, IFITM3, BST2, IFITs, and TRIM (18, 19).

**Figure 3:**
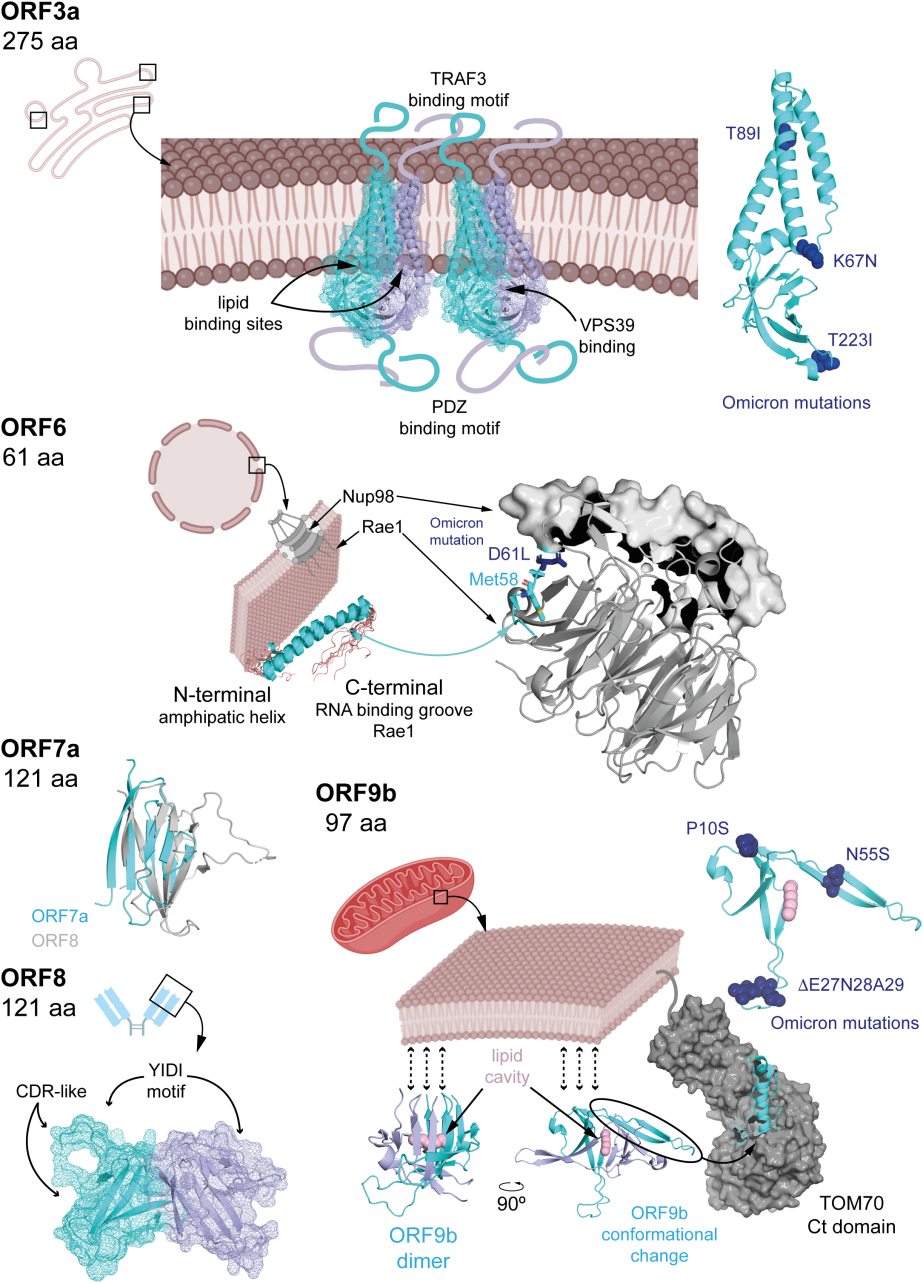
Known APs structural features. ORF3a is a transmembrane dimeric protein with long flexible regions extending on both sides of the membrane. Its structure has been determined from nanodisc-embedded samples using cryo-electron microscopy samples. Several binding motifs have been identified through structural data, sequence analysis, and mutagenesis studies. The structure of ORF6 has been resolved by NMR, and its interaction mechanism with rae1 has been elucidated using macromolecular crystallography, denoting the relevance of a conserved Met. ORF7a and ORF8 possess Ig-like domains, whose structures have also been determined by macromolecular crystallography. ORF9b exhibits an enriched b-strand fold containing a hydrophobic pocket capable of accommodating lipids, potentially anchoring dimers to membranes. The structure of ORF9b in complex with TOM70 revealed a dramatic conformational change in which two b-strands rearrange into a helix, suggesting possible folding transitions depending on the targeted host protein. In the figure, APs structures are shown in cyan cartoon representation (also displaying mesh representation for ORF3a and ORF8), with dimeric forms in light blue. Omicron mutations (**Figure 5**) are highlighted in dark blue and stick or sphere representations, when possible (i.e. the corresponding region in the protein is structured). Host-interacting proteins are in grey, either in cartoon (Rae1) or surface (Nup98, TOM70) representation, and lipids highlighted in pink. PDBs 6xdc (ORF3a), 7vph (Nup98-Rae1-ORF6), 7ci3 (ORF7a), 7jtl (ORF8), 7ye8 (ORF9b) and, 7dhg (TOM70-ORF9b) were used to create the corresponding figures in Pymol.

Like many viruses (20), SARS-CoV-2 has evolved multiple strategies to interfere with IFN-I signalling. Several APs disrupt different steps along this pathway (**Figure 2**). Once IFN-α/β is released, some APs can impair the signalling cascade at steps taking place before the nuclear translocation of ISGF3. This is the case of ORF3a, which activates SOCS1, a JAK/STAT repressor promoting JAK2 degradation, thereby reducing STAT1 phosphorylation, leading to reduced transcription of multiple ISGs (2, 21–23). ORF3a, is a 275 amino acids transmembrane protein (**Figure 3**) that comprises an N-terminal ectodomain (residues 1–39), containing a signal peptide and a flexible TRAF3-binding motif; three transmembrane domains (residues 40–128) with conserved lipid-binding sites; and a cytosolic C-terminal β-sandwich domain (residues 145–235) harbouring a YXXF sorting motif that interacts with VPS39 of the HOPS complex (24, 25). This is followed by a flexible C-terminal tail (residues 239–275) containing a PDZ-binding motif (26). Cryo-EM studies show that ORF3a forms homodimers that can further oligomerize through the transmembrane region (27, 28). ORF3a localizes to the plasma membrane and to the membranes of various intracellular organelles, including endosomes, lysosomes, ER, Golgi, peroxisomes, and autophagosomes, which is regulated by three caveolin-binding motifs (residues 69 – 77, 107–114 and 141 – 149) (27, 29). It remodels membranes to form 3a Dense Body (3DB), derived from the trans-Golgi network and early endosomes (30). Initially described as a viroporin disrupting ion flux, its proposed role remains controversial as patch-clamp studies failure to detect ion conductance by ORF3a pores (27, 28). Consequently, the dynamic subcellular distribution of ORF3a facilitates extensive interference with host cellular pathways, reflecting its complex and multifunctional role in viral pathogenesis.

The putative ORF3b (22 amino acids) strongly inhibits IRF3 nuclear translocation and IFN-β production upon overexpression in HEK293 and A549 cell lines, more potently than its SARS-CoV homolog. However, its precise mechanism remains unclear (31). Infection-related ORF3c (41 residues) interferes with MAVS signalling at mitochondria by interacting with MAVS and PGAM5, a regulator of mitochondrial dynamics. ORF3c expression leads to cleavage of MAVS C-terminal domain, likely via caspase-3 activation, reducing IFN-β levels (32, 33).

Downstream, ORF6 (61 amino acids) impairs both IFN-α/β production and ISG activation. It interacts with the nuclear import machinery: by binding KPNA2, ORF6 inhibits IRF3 and STAT1 nuclear translocation (2, 34), while association with the Nup98-Rae1 nucleopore complex blocks ISGF3 nuclear entry (35). ORF6 is a three domains protein (**Figure 3**) including N- and C-terminal domains separated by long straight helix (residues 11-45). The N-terminal domain folds into an amphipathic helix responsible of membrane association and that promote transient protein-protein interactions contributing to antiparallel dimerization. The flexible C-terminal domain binds Rae1 with nanomolar affinity competing with host mRNAs for transport through the nucleopore complex and hence, inhibiting their nuclear export (including IRF3 and ISG3). To gain high binding affinity uses a similar strategy to M protein of VSV and ORF10 Murine Gammaherpesvirus 68 (36, 37), with a conserved Met58 residue that inserts into a hydrophobic pocket on Rae1; mutation Met58Arg abolishes Nup98 interaction but not KPNA2 binding (35, 38–40). These interactions allow ORF6 to block nuclear import of key IFN-I signalling factors.

ORF7a (121 amino acids) consists of an N-terminal signal peptide, an Ig-like β-sandwich ectodomain, a transmembrane domain, and a short C-terminal tail with an ER retention signal (41) (**Figure 3**). It suppresses STAT2 phosphorylation via K63-linked polyubiquitination at Lys119 by the ER-associated RNF121 E3 ligase, inhibiting ISG expression such as ISG56, IFITM1, and OAS (2, 42).

The roles of ORF7b (43 amino acids) and ORF8 (121 residues) in IFN-I modulation are controversial. ORF7b is a membrane-associated protein unique to SARS-CoV-2. It adopts an α-helical structure with a leucine zipper motif enabling multimerization and interaction with host proteins. ORF7b forms tetramers interacting with MAVS, disrupting MAVS-TRAF6 interaction and IFN-β production (43). Some studies have found that ORF7b inhibits STAT1/2 phosphorylation and ISG transcription (2, 44), while others report minimal effects (45). A truncated ORF7b variant (Δ382) loses IFN suppression ability and correlates with milder disease (45, 46). ORF8 (121 amino acids) is structurally similar to ORF7a (**Figure 3**) with an immunoglobulin-like fold. However, ORF8 includes two novel dimerization interfaces and several unique features. Particularly, it includes a glycosylation site (Asn78), a lipid binding pocket and three functional loops resembling antibody complementarity determining regions (47–49). These features are strategically positioned to facilitate interactions with host proteins. Variants with deletions or mutations such as Δ382 or Leu84Ser modulate IFN-I responses and disease severity (50–52). ORF8 interacts with HSP90B1, potentially disrupting IRF3 activation and nuclear translocation, although its inhibitory effect on IFN-I signalling appears weaker than ORF6 (34, 53).

ORF9b (97 amino acids) acts upstream of IFN-I expression by binding to the C-terminal domain of the mitochondrial translocase TOM70. This interaction disrupts mitochondrial recruitment and phosphorylation of TBK1, thereby suppressing IFN-I production (54–59).ORF9b exists as a homodimer with a hydrophobic cavity capable of accommodating lipids and hence, potential binding to membranes. It undergoes a dramatic conformational change upon TOM70 binding (from β-stand to helix, **Figure 3**) (54, 60, 61). The mechanism underlying this conformational shift remains unclear. In fact, the functionality of ORF9b is dependent on its ability to dimerize. Small molecules that inhibit the homodimerization of ORF9b have been shown experimentally to disrupt ORF9b–ORF9b interactions, promoting mitochondrial eviction of ORF9b, inhibiting ORF9b-induced activation of caspase-1, and restoring type I interferon (IFN-I) signalling (59). Also, ORF9b harbours multiple phosphorylation sites, a specific phosphorylation signature that may act as a switch for structural rearrangement (54, 61). Host E3 ubiquitin ligases mediate the proteasomal degradation of ORF9b, a process counteracted by the deubiquitinating enzyme USP29, regulating ORF9b levels and IFN-I suppression (57, 58). Therefore, the impact of ORF9b on IFN-I signalling appears to depend on a dynamic balance between its structural inhibition of mitochondrial signalling and its post-translational regulation through the ubiquitin-proteasome system.

Finally, putative ORF10 (38 amino acids) promotes MAVS degradation through interaction with the CUL2^ZYG11B^ E3 ubiquitin ligase complex, mediating protein ubiquitination and subsequent proteasomal degradation (13, 62). In this context, ORF10 upregulates NIX-expression, a mitochondrial receptor known to interact with LC3B, a key component of the autophagosome membrane. This interaction facilitates mitophagy, leading to MAVS degradation and IFN-I suppression (13, 63). Despite being classified as a putative AP (**Figure 1A**), high levels of *ORF10* transcripts have been reported in patients with severe COVID-19, whereas protein levels were not included in the analysis (64). Thus, conclusions regarding ORF10’s involvement in IFN-I modulation should be interpreted with caution.

### 2.2 Inflammatory pathways and cytokine induction

**Figure 4:**
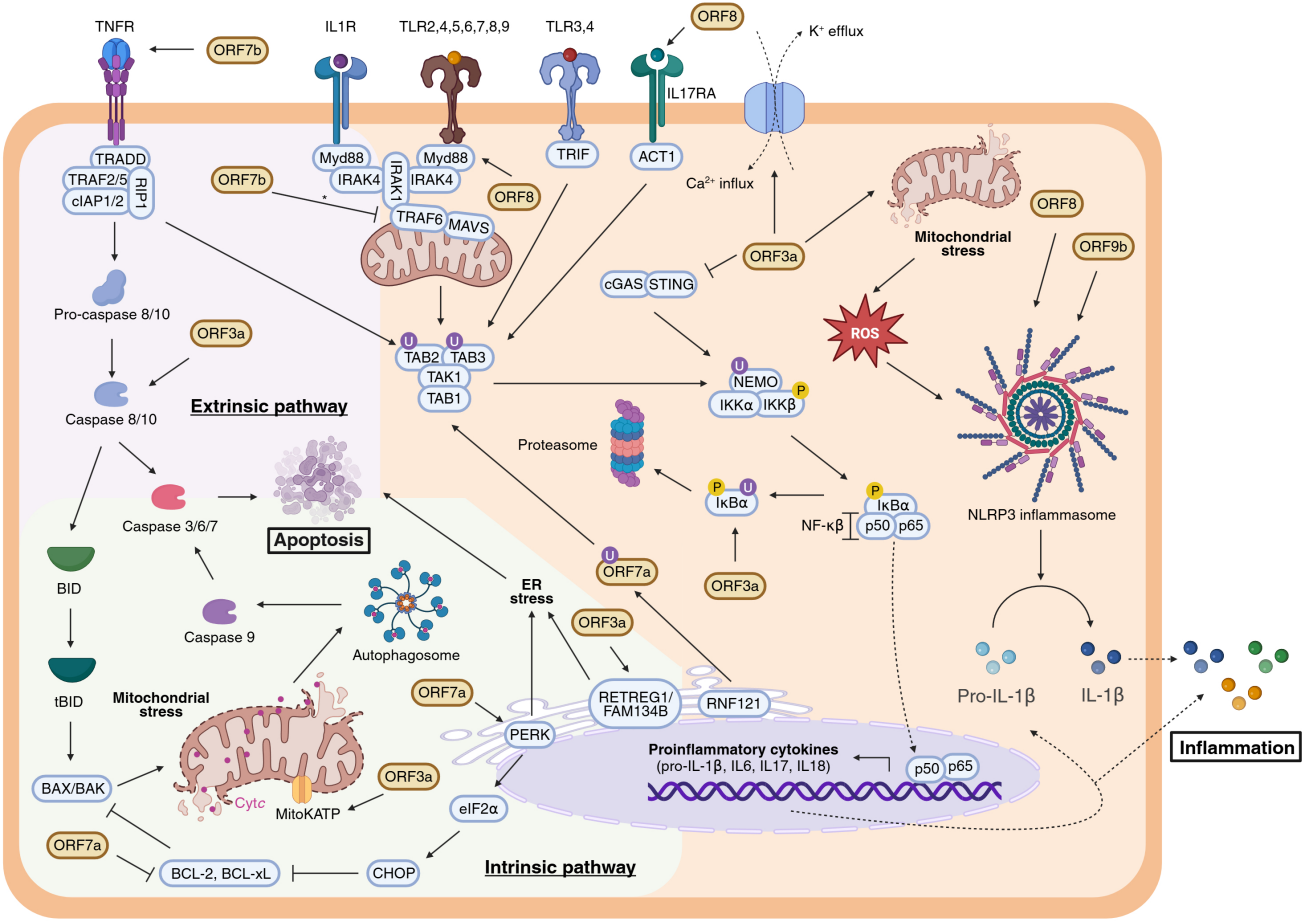
Modulation of the apoptotic pathways and the inflammatory response by SARS-CoV-2 APs. Extrinsic and intrinsic apoptotic pathways are respectively represented within a lilac and green background, whereas inflammatory response (NF-κβ signalling and NLRP3 inflammasome activation) is coloured in orange. Inconclusive results are indicated with an asterisk (*). Figure created with BioRender.com.

Beyond the IFN-I response, viral infections profoundly alter proinflammatory cytokine expression. Viral components and cytokines activate pattern recognition receptors such as Toll-like receptors (TLRs), tumour necrosis factor receptor (TNFR), interleukin receptors (IL1R, IL17RA), triggering a signalling cascade via TAK1 activation. TAK1 is recruited and activated through ubiquitination mediated by TAB1/2/3 and TRAF6. Activated TAK1 phosphorylates IKKβ, which in turn phosphorylates the inhibitor IκBα. Phosphorylated IκBα is degraded, freeing NF-κB (p50-p65) to translocate into the nucleus and promote transcription of proinflammatory cytokines including IL-1β, IL-6, and TNFα. The phosphorylation and degradation of IκBα thus constitute a critical regulatory step in NF-κB–mediated inflammation (65, 66) (**Figure 4**).

While NF-κB activation initiates inflammation, a second mechanism involves inflammasome assembly. Pro-IL-1β, an inactive cytokine precursor, requires proteolytic cleavage by caspase-1 for activation. The NLRP3 inflammasome—a multiprotein complex—is assembled upon detection of various pathogen-associated molecular patterns (PAMPs) and damage-associated molecular patterns (DAMPs), including K^+^ efflux, Ca²^+^ influx, and reactive oxygen species (ROS) release. These signals trigger caspase-1 activation and subsequent maturation of IL-1β, amplifying the inflammatory response (67) (**Figure 4**).

Among SARS-CoV-2 APs, ORF3a plays a pivotal role in modulating inflammation. ORF3a promotes NF-κB activation by inducing IκBα phosphorylation and degradation in HEK293T and A549 cells (68). However, the exact molecular target through which ORF3a triggers IκBα degradation remains undefined. Notably, ORF3a’s effect on NF-κB is independent of the cGAS-STING pathway. Indeed, co-expression of ORF3a with cGAS and STING reduces NF-κB activation compared to cGAS-STING alone, likely via direct interactions with STING’s N- and C-terminal domains and intracellular co-localization (69, 70). Moreover, ORF3a induces K^+^ efflux, a well-known inflammasome activator, leading to increased NLRP3, caspase-1 activation, and cleavage of IL-1β (68, 71). It further promotes TRAF3-dependent ubiquitination that enhances NLRP3 inflammasome activation (72). ORF3a is also implicated in mitochondrial damage and ROS production, culminating in inflammatory cell death or pyroptosis (3, 73–75). Collectively, these findings identify ORF3a as a critical mediator of inflammatory signaling, acting through both NF-κB activation and inflammasome stimulation. Thus, it is plausible to suggest ORF3a as a central contributor to the cytokine storm observed in severe COVID-19 cases (76, 77).

Other APs, notably ORF7a and ORF7b, modulate inflammatory pathways. Unlike its SARS-CoV homolog, SARS-CoV-2 ORF7a binds efficiently to CD14^+^ monocytes via its Ig-like ectodomain and downregulates HLA-DR/DP/DQ molecules, potentially impairing antigen presentation and disrupting immune homeostasis (41). Ubiquitination at Lys119 by the ER-associated E3 ligase RNF121 enables ORF7a’s interaction with TAK1, facilitating TAK1 activation and amplifying inflammatory signalling (78). Consistently, ORF7a expression correlates with increased proinflammatory cytokine levels (41, 44, 79). Conversely, ORF7b’s role is less clear. One report shows that ORF7b inhibits proinflammatory cytokines by disrupting TRAF6-MAVS interactions, hindering TAK1-TAB complex assembly and cytokine expression in HEK293T cells (43). In contrast, another study reports that ORF7b induces the expression of proinflammatory cytokines such as IL-8 and CXCL1 in A549 cells (44), indicating possible context-dependent effects.

ORF8 is a secreted AP frequently detected in serum of COVID-19 patients (80–82). Nonetheless, its mechanism of action in modulation the inflammatory response remains unclear and appears to be dependent on cell-type specificity. It mimics IL-17 by binding IL17RA, activating a broad proinflammatory cytokine response in human CD14^+^ monocytes, dendritic cells, and epithelial cell lines (83–87). ORF8 undergoes N-glycosylation at Asn78 via the classical Golgi secretory pathway (86); glycosylation reduces its affinity for IL17RA and dampens its proinflammatory activity, whereas unglycosylated ORF8, secreted via ER-Golgi intermediate compartment vesicles, induces stronger NF-κB activation (84, 86, 88). However, ORF8 does not mimic IL-17 or IL-1 in human or murine macrophages and may inhibit NF-κB signalling downstream of IL-1R by interacting with MyD88, revealing complex and cell-type specific roles (89). Additionally, ORF8 may enter peripheral blood mononuclear cells via phagocytosis. Importantly, ORF8 is also implicated as a potent inflammasome activator, though further studies are needed to clarify these mechanisms (81).

ORF9b has been described as a potent inhibitor of NF-κB signalling by binding the NEMO subunit of the IκB kinase complex, preventing its ubiquitination and assembly, thereby suppressing proinflammatory cytokine expression such as TNFα and IP-10 in HEK293T cells (90). Nonetheless, recent evidence indicates that ORF9b can also promote NLRP3 inflammasome activation in A549 and THP-1 cells, with inhibitors of ORF9b dimerization reversing this proinflammatory phenotype (59). The structural flexibility of ORF9b, including conformational changes upon TOM70 binding, suggests it may adopt distinct forms to modulate multiple host pathways independently, contributing to immune dysregulation.

In addition to acute inflammation, SARS-CoV-2 infection also induces pulmonary fibrosis in severe COVID-19 infections (91). Individual expression of ORF6, ORF8, ORF9b, and ORF9c promotes pro-fibrotic processes in lung-derived cells by upregulating IL-11 signalling. This upregulation is accompanied by altered expression of extracellular matrix remodelling genes such as WNT5A and signalling mediators including STAT3 and TGF-β. Functional assays confirm that this IL-11-driven response induces profibrotic cellular phenotypes, which are recapitulated in SARS-CoV-2-infected lung cells and patient lung tissue. These observations implicate these APs as contributors to the inflammatory and fibrotic lung pathology characteristic of severe COVID-19 (92).

## 3 Apoptosis

Apoptosis is a regulated form of cell death essential for tissue homeostasis and the elimination of damaged or unwanted cells. It is executed via three main pathways: the extrinsic (death receptor-mediated), intrinsic (mitochondria-mediated), and endoplasmic reticulum (ER) stress/unfolded protein response (UPR)-mediated pathways. All converge on the activation of caspases, a family of cysteine-aspartic proteases responsible for cellular dismantling (93). The extrinsic pathway is initiated by death receptors such as Fas and TNFR1, leading to the formation of the death-inducing signalling complex (DISC) and activation of caspase-8. In turn, caspase-8 activates effector caspases or amplifies the signal through the intrinsic pathway via Bid cleavage (94). The intrinsic pathway responds to internal stress signals and is tightly regulated by Bcl-2 family proteins that control mitochondrial outer membrane permeabilization (MOMP) and cytochrome c release, activating caspase-9 and downstream effectors. ER stress activates UPR sensors (PERK, IRE1, ATF6), and prolonged stress induces apoptosis through transcription factors such as ATF4/CHOP or JNK (93).

SARS-CoV-2 has evolved multiple strategies to manipulate apoptotic pathways for its advantage, either by inducing apoptosis in immune cells to evade host defences or by inhibiting apoptosis in infected cells to prolong viral replication. This dual regulation is both stage- and cell type–dependent, reflecting the virus’s ability to fine-tune host cell survival according to its replication needs (95). Several SARS-CoV-2 APs have been implicated in modulating apoptosis through diverse mechanisms (**Figure 4**).

ORF3a is the most extensively characterized AP linked to apoptosis regulation. Evidence shows that ORF3a activates both extrinsic and intrinsic apoptotic pathways. It induces caspase-8 activation, leading to Bid cleavage, mitochondrial cytochrome c release, and subsequent apoptosis. Specific inhibitors of caspase-8 and caspase-9 markedly reduce ORF3a-induced apoptosis, underscoring the involvement of both pathways (96). Furthermore, ORF3a disrupts mitochondrial homeostasis by modulating the mitochondrial ATP-sensitive potassium channel (MitoKATP), resulting in mitochondrial dysfunction and apoptotic induction (74). In the ER, ORF3a induces RETREG1/FAM134B-dependent reticulophagy via the HMGB1-BECN1 pathway, causing ER stress and inflammatory responses that sensitize cells to early ER stress-related apoptotic phenotypes (97). *In vivo* studies using mice transfected with ORF3a reveal extensive lung tissue damage, increased apoptotic cell death, and enhanced inflammation, providing direct evidence of ORF3a’s role in apoptosis and COVID-19 pulmonary pathology (74). Taken together, these observations position ORF3a as a central mediator of SARS-CoV-2–induced cytotoxicity, mechanistically linking mitochondrial and ER stress–mediated apoptosis to the tissue injury and inflammation characteristic of severe COVID-19.

ORF7a promotes apoptosis by recruiting the anti-apoptotic protein Bcl-xL to the ER through its exposed C-terminal residues Lys117 and Lys119, impairing Bcl-xL’s protective function and triggering the PERK-eIF2α-CHOP ER stress pathway. This activation initiates the intrinsic apoptotic cascade via BAK/BAX induction and cytochrome c release. Ubiquitination of ORF7a at Lys117/Lys119 disrupts this interaction, attenuating ER stress activation and protecting cells from apoptosis (98).

ORF7b induces apoptosis primarily through tumour necrosis factor alpha (TNF-α) signalling. Overexpression of ORF7b in cell models (e.g., HEK293T, Vero E6) enhances phosphorylation of IRF3 and STAT1, both of which translocate independently to the nucleus and promote TNF-α secretion. TNF-α then binds its receptor TNFR1, activating caspase-8 and subsequently caspases-3 and -9, culminating in apoptosis (46). In lung epithelial cells, ORF7b also triggers ferroptosis; inhibitors of both apoptosis and ferroptosis block ORF7b-induced cell death. ORF7b increases expression of the transcription factor c-Myc, a pivotal regulator of cell death pathways, and c-Myc depletion markedly reduces apoptosis, ferroptosis, and lung damage in murine models (99).

Conversely, ORF9b does not directly induce apoptosis but influences apoptotic pathways by targeting mitochondrial components. As mentioned, the interaction of ORF9b with TOM70 inhibits downstream activation of TBK1 and IRF3, which are transcription factors required for pro-apoptotic proteins such as Noxa and Puma (**Figure 2**) (100, 101). This interference may delay apoptosis, allowing extended viral replication. Additionally, ORF9b, together with NSP4, induces the formation of macropores in the mitochondrial outer membrane, facilitating the release of mitochondrial DNA (mtDNA) into the cytosol and extracellular space. This mtDNA release is both pro-inflammatory and pro-apoptotic and has been linked to severe COVID-19 outcomes (102).

Together, these findings illustrate the dual nature of SARS-CoV-2’s apoptotic regulation (**Figure 4**). By selectively activating or suppressing programmed cell death depending on the cellular context and stage of infection, SARS-CoV-2 balances two opposing needs: preserving host cell viability to sustain replication or promoting cell death to evade immune surveillance and exacerbate tissue damage. This finely tuned, double-edged modulation of apoptosis underscores the virus’s remarkable ability to exploit host cell death pathways as both a replicative and pathogenic strategy.

## 4 Metabolic reprogramming

Metabolic reprogramming refers to the capacity of pathogens to alter host cellular metabolism, thereby creating a favourable environment for viral replication and dissemination. Viruses commonly manipulate key metabolic pathways including glycolysis, fatty acid synthesis, glutaminolysis, and mitochondrial bioenergetics (103–105). SARS-CoV-2 profoundly disrupts systemic and cellular homeostasis, impairing organ function and contributing to COVID-19 severity (106, 107). Notably, infection triggers extensive metabolic remodelling in immune cells, particularly monocytes in high-glucose conditions, which compromises T-cell function and promotes lung epithelial cell death — potentially explaining worse outcomes in patients with metabolic comorbidities such as type 2 diabetes (108, 109).

A hallmark of SARS-CoV-2-induced metabolic remodelling is a shift toward aerobic glycolysis, reminiscent of the Warburg effect in cancer cells (108). The virus enhances glycolytic flux and alters redox and energy balance to meet its replicative and biosynthetic demands (4, 110). This metabolic switch is partly driven by stabilization of hypoxia-inducible factor-1 alpha (HIF-1α), a master regulator of glycolytic gene expression and inflammatory cytokine production (75, 108, 111). Elevated mitochondrial reactive oxygen species (ROS) in infected cells stabilize HIF-1α, promoting its nuclear translocation and dimerization with HIF-1β. This complex upregulates glucose transporters (GLUT1, GLUT3, GLUT4), key glycolytic enzymes (HK2, PFK1, PKM2, LDHA), and proinflammatory cytokines such as IL-1β (75, 108, 112). The resulting increase in glucose uptake and lactate production further stabilizes HIF-1α, establishing a feed-forward loop that amplifies inflammation and viral replication. Elevated lactate suppresses MAVS signalling, thereby impairing antiviral defences (113). By diverting host metabolism toward glycolysis, SARS-CoV-2 ensures rapid ATP and biosynthetic precursor production, favouring viral genome replication, protein synthesis, and virion assembly, an evolutionarily advantageous strategy shared by many viruses (114).

**Figure 5:**
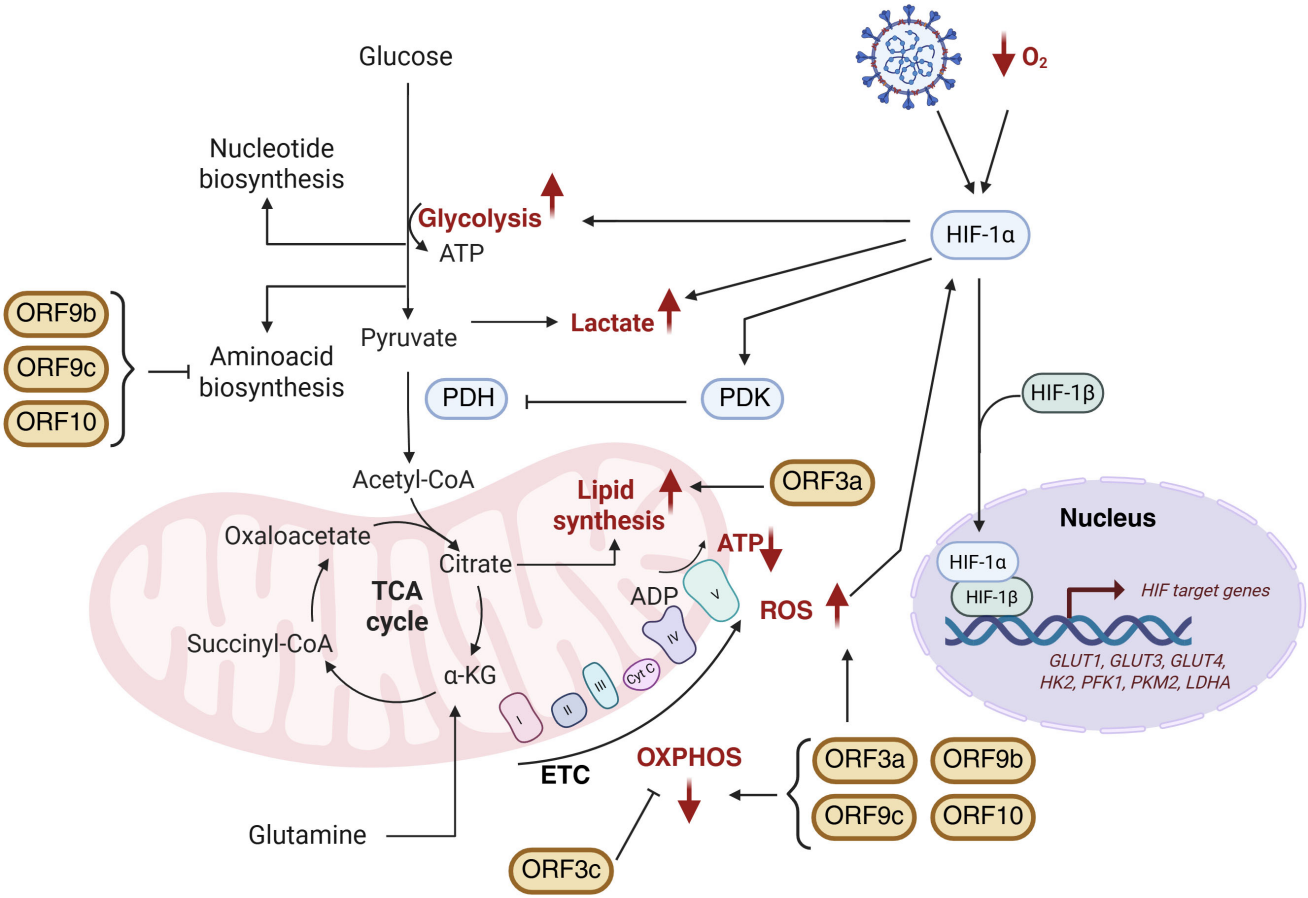
Schematic representation of SARS-CoV-2 APs-induced metabolic reprogramming. Mitochondrial alterations including HIF-1α stabilization, glycolysis activation, lactate production as well as modifications in TCA cycle, oxidative phosphorylation (OXPHOS) and lipid and amino acid biosynthesis are represented. Metabolic changes highlighted in red correspond to those observed during SARS-CoV-2 infection. Figure created with BioRender.com.

Emerging evidence implicates SARS-CoV-2 APs in metabolic reprogramming (**Figure 5**). Among these, ORF3a is a major disruptor, altering mitochondrial dynamics and metabolic fluxes. ORF3a induces mitochondrial ROS, enhancing HIF-1α stabilization and promoting glycolysis and subsequent lactate production (72, 75, 108). It compromises mitochondrial integrity by disrupting cristae structure and inducing fragmentation, accompanied by reduced mitochondrial membrane potential (ΔΨm) (3). These changes correlate with oxidative stress, impaired respiration, and decreased ATP production, characterized by reduced oxygen consumption rate (OCR), dysfunction of complexes I and V, and oxidative damage to mitochondrial proteins including ATP synthase (ATP5A1) and ATP/ADP translocase (SLC25A4) (115). Electron transport chain (ETC) disruption is exacerbated by calcium influx and mitochondrial permeability transition pore (mPTP) opening (74). Transcriptomic analyses reveal ORF3a-induced downregulation of mitochondrial genes across ETC complexes I, III, IV, and V, as well as key cristae regulators OPA1, MIC60 (IMMT), and TOMM40 (3).

In addition to mitochondrial effects, ORF3a inhibits autophagy by interacting via its C-terminus with VPS39, disrupting VPS39’s binding to RAB7A and the SNARE protein STX17. Since RAB7A-HOPS complex formation is essential for autophagosome-lysosome fusion, ORF3a-mediated sequestration of VPS39 impairs this process, leading to accumulation of unfused autophagosomes and preventing viral degradation. Beyond avoiding degradation, the accumulation of autophagosomes provides SARS-CoV-2 with double-membrane vesicles that act as protected replication niches and shielded from cytosolic immune sensors, thereby enhancing viral replication efficiency (116). Moreover, ORF3a induces lipid droplet accumulation during infection, facilitating viral replication — a feature distinguishing SARS-CoV-2 from SARS-CoV (117). Genome-scale metabolic modelling (GSMM) supports these findings, revealing that ORF3a promotes increased lipid metabolism alongside suppression of oxidative phosphorylation (OXPHOS), amino acid biosynthesis, and one-carbon metabolism (3).

ORF3c is a newly identified mitochondrial modulator that localizes specifically to mitochondria and shifts cellular fuel usage from glucose toward fatty acid oxidation, enhancing OXPHOS. This rewiring increases ROS generation and causes autophagy blockade via impaired lysosomal acidification. Notably, this autophagy inhibition is unique to SARS-CoV-2 ORF3c, as the homolog from batCoV RaTG13 lacks this function due to amino acid differences at Arg36 and Lys40 (118).

In contrast, ORF9b, ORF9c, and ORF10 share a metabolic profile characterized by downregulation of amino acid and fatty acid metabolism and impairment of mitochondrial respiration. These APs suppress OXPHOS without inducing compensatory glycolysis. Functional assays confirmed this effect by showing reduced basal and compensatory glycolytic rates in cells expressing these proteins. Conversely, ORF3a-expressing cells maintain glycolytic flux comparable to controls, suggesting activation of glycolytic rescue mechanisms to preserve ATP levels. Among these, ORF9b causes the most severe mitochondrial dysfunction, markedly decreasing basal and maximal respiration, spare respiratory capacity, and ATP production. ROS accumulation is notably higher in ORF9c- and ORF10-expressing cells, correlating with diminished expression of antioxidant enzymes such as ALDH7A1 (3).

The coexistence of these distinct metabolic effects likely reflects a viral division of labour, enabling SARS-CoV-2 to adapt to diverse cellular environments and infection stages, sustaining replication in metabolically active cells while driving energy collapse and immune suppression in others. The ability of SARS-CoV-2 APs to reprogram host metabolism unveils novel therapeutic opportunities. Persistent mitochondrial dysfunction and metabolic imbalance induced by APs may contribute to post-acute sequelae, including Long COVID, underscoring the clinical relevance of targeting virus-induced metabolic rewiring. Computational analyses using the qMTA algorithm identified phospholipase D2 (PLD2) as a promising target to reverse ORF3a-driven metabolic perturbations. Pharmacological inhibition of PLD2 with CAY10594 restored mitochondrial morphology and partially reversed altered metabolic fluxes, particularly within lipid metabolism pathways (3). Together, these findings highlight the therapeutic potential of targeting virus-induced metabolic rewiring to limit viral replication and mitigate COVID-19 pathogenesis (**Figure 5**).

## 5 Impact of emerging mutations in SARS-CoV-2 APs on host responses

**Figure 6:**
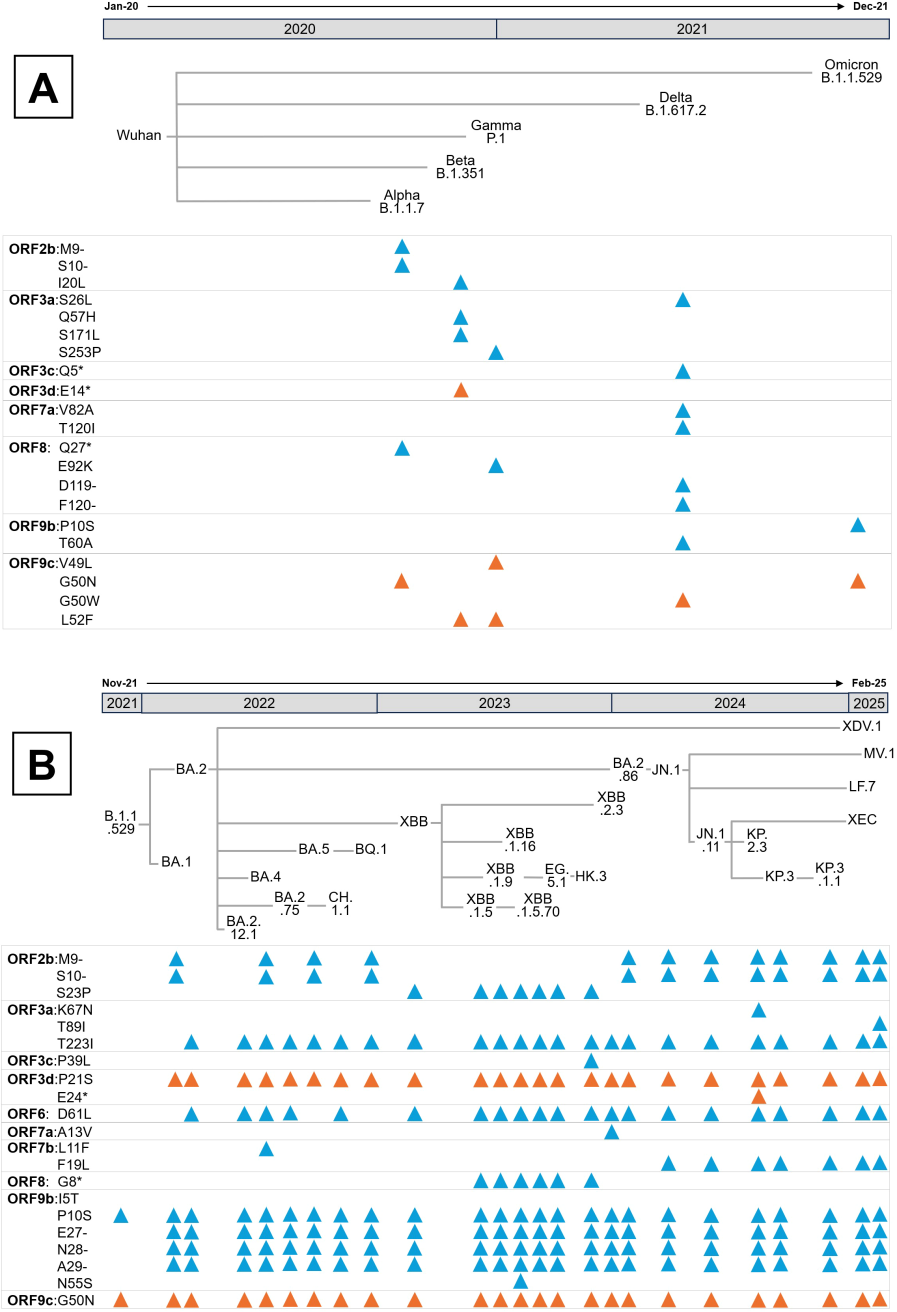
Evolution of SARS-CoV-2 APs since the beginning of the pandemic. **(A)** Mutations presented in the APs of the five Variants of Concern (VOCs), designated by the WHO as Alpha, Beta, Gamma, Delta and Omicron. According to the Pango nomenclature, the parental lineage of each VOC is also indicated. **(B)** Mutations present in the major Omicron lineages from its emergence in November 2021 to February 2025. In both panels, blue triangles represent mutations in coding APs, while orange ones indicate mutations in non-coding APs. Mutations and phylogenetic relationships between lineages were obtained from CoVariants (120).

Emerging mutations in SARS-CoV-2 APs have the potential to modulate host immune responses. However, until the emergence of the Omicron variant in late 2021, SARS-CoV-2 APs exhibited minimal evolutionary changes. Rather than indicating weak immune selective pressure, this evolutionary stasis likely reflects strong purifying selection, suggesting that APs functions were already finely tuned for viral fitness (119). This is exemplified by the first four Variants of Concern (VOCs)—Alpha, Beta, Gamma (2020), and Delta (2021)—which exhibited minimal changes in APs sequences (**Figure 6A**). Therefore, SARS-CoV-2 APs protein sequence were nearly optimal for maintaining virus transmissibility. The subsequent diversification observed in Omicron may thus represent a shift in the virus’ adaptive landscape, enabling exploration of new functional or immune-evasive strategies (119, 120).

Several conserved mutations in overlapping genes, whose effects on host modulation remain unexplored, are depicted in **Figure 6A** and **Figure 6B**. For instance, the ORF2b Δ9–10 deletion, first identified in Alpha and retained in most Omicron lineages, results in the loss of His69 and Val70 in the N-terminal domain of the S protein, a modification known to enhance infectivity (121). Even though ORF2b encodes a small 39-amino-acid protein confirmed to be expressed during infection (122), its function remains unknown, warranting further investigation into the biological consequences of both ORF2b isoforms.

Mutations in ORF3a also demonstrate varied functional impacts. The Ser26Leu substitution in Delta occurs in a highly variable region among sarbecoviruses and is considered functionally negligible (72). Similarly, the Beta variant’s Gln57His substitution, located at the dimerization interface, retains comparable Ca²^+^ permeability, apoptotic induction, and proinflammatory capacities in cell models (123). Additionally, the Ser253Pro substitution in Gamma does not appear to affect ORF3a’s oligomerization or localization (124). Conversely, the Beta-specific Ser171Leu mutation abolishes ORF3a-driven lipid-droplet accumulation, a phenotype linked to viral replication efficiency (117). Notably, the Omicron lineage’s Thr223Ile substitution (**Figures 3**, **6B**) disrupts ORF3a interaction with VPS39, impairing lipid-droplet formation and reducing replication, consistent with Omicron’s lower pathogenicity (117, 125). In HK2 cells this variant also shifts ORF3a localization from lysosomes to Golgi and ER compartments and, paradoxically, enhances proinflammatory cytokine production despite Omicron’s attenuated clinical severity (126). Two non-mutually exclusive explanations could account for this *in vitro* paradox. Firstly, ORF3a is known to trigger endomembrane stress and inflammatory signalling (25). Thr223Ile-driven re-localization to ER/Golgi may increase ER stress and activate innate signalling pathways such as NF-kB or NLRP3, amplifying cytokine responses even as viral replication declines. Secondly, disruption of the ORF3a-VPS39 axis and consequently autophagy/lysosomal defects may alter cellular handling of viral RNA or proteins. This effect will, in turn, heighten exposure of pathogen-associated molecular patterns to cytosolic sensors, thereby potentiating cytokine induction, a response pronounced *in vitro* but moderated *in vivo* by tissue-level regulation (25). Both hypotheses align with studies linking ORF3a to lysosomal trafficking, inflammasome activation, and lipid-droplet dependent replication, underscoring the need for mechanistic analysis using ER-stress markers, autophagy flux assays, and innate sensor knockdowns to delineate these effects.

Additional mutations in Putative APs include the ORF3d Pro21Ser substitution emerging in BA.1 and BA.2 Omicron lineages. Ribosome profiling indicates that only a shorter ORF3d isoform (ORF3d-2) is translated during infection (122). Premature stop codons in the Beta variant and KP.2.3 Omicron lineage affect the full-length ORF3d but not ORF3d-2, reinforcing the latter’s relevance *in vivo*.

The BA.2 Omicron lineage also acquired the ORF6 Asp61Leu substitution, conserved in most subsequent Omicron lineages except BA.5 and BQ.1 (**Figure 6B**). ORF6 primarily antagonizes the IFN-I response by inhibiting nuclear import of IRF3 and STAT1/2 (**Figure 2**). Interestingly, the Asp61Leu substitution increases IFN-β and ISG expression, likely by reducing ORF6 interaction with the Rae1-Nup98 complex, as this mutation increases the hydrophobicity of ORF6’s acidic C-terminal domain essential for this interaction (35, 39, 40, 127). Therefore, Omicron ORF6 may diminish antiviral responses triggered by its Wuhan counterpart, potentially resulting in reduced pathogenicity.

The ORF7b Phe19Leu mutation, emerging in the JN.1 Omicron lineage (early 2024), has been linked to enhanced viral replication and spread *in vitro* and in Syrian hamsters (128). Given that ORF7b contains a leucine-zipper motif, this substitution may stabilize the association of monomers with membranes, influencing its immunomodulatory roles (**Figures 2**, **4**).

Most other APs mutations in Omicron lack conservation and functional characterization. Notably, except for XBB.1.5 and XBB.1.5.70 lineages, ORF8 remains largely wild type in Omicron. However, truncations of ORF8, observed in Alpha and those XBB sublineages (**Figures 6A**, **B**), disrupt its Ig-like domain, leading to increased lung inflammation in mouse models relative to wild-type ORF8 infection (125, 129). The Glu92Lys substitution in Gamma yields an intermediate inflammatory phenotype, likely due to altered monomer interface charge (129), suggesting that ORF8 may attenuate SARS-CoV-2 pathogenicity. This hypothesis is consistent with its conservation in less virulent Omicron variants.

ORF9b has acquired two notable mutations in Omicron: Pro10Ser in the parental lineage and a Δ27–29 deletion independently in BA.1 and BA.2, both retained in current lineages (**Figure 6B**). Given that ORF9b’s N-terminal interaction with NEMO suppresses NF-κB signaling (90), and its structural flexibility upon TOM70 binding (130), these mutations could affect IFN-I antagonism and inflammation regulation. Conversely, the ORF9b Ile5Thr mutation in recent XBB sublineages showed minimal effects on IFN suppression and replication, correlating with its disappearance in later lineages (131).

Finally, conserved mutations in overlapping genes such as ORF9c Gly50Asn — linked to the N protein’s Arg203Lys-Gly204Arg double substitution that enhances viral infectivity and recombination (132, 133) — require further functional studies. ORF9c is known to disrupt mitochondrial metabolism and induce profibrotic IL-11 signalling in lung cells (3, 92), underscoring the necessity to clarify the impact of these mutations on SARS-CoV-2 pathogenesis.

In conclusion, evolution of SARS-CoV-2 APs in the Omicron variant appears to have played a pivotal role in its attenuated virulence, establishing a new phase in virus-host coevolution. Several conserved mutations, including ORF3a Thr223Ile, ORF6 Asp61Leu, ORF7b Phe19Leu, and ORF9b Pro10Ser along with the ORF9b Δ27–29 deletion, are maintained across Omicron sublineages, suggesting selective advantages that fine-tune virus-host interactions. Collectively, these alterations seem to recalibrate viral replication, immune evasion, and inflammatory potential, shifting SARS-CoV-2 toward a state of enhanced human adaptation and reduced pathogenicity.

## 6 Concluding remarks and future perspectives

APs have emerged as critical modulators of SARS-CoV-2 biology, orchestrating host-virus interactions beyond the functions of the structural and replicase proteins. Although non-essential for viral replication, APs profoundly influence immune evasion, cellular stress responses, and pathogenicity, shaping the clinical course and transmissibility of COVID-19. Understanding their roles is therefore fundamental for deciphering mechanism underlying SARS-CoV-2 virulence and persistence. In this review, we synthetize current knowledge of how SARS-CoV-2 APs modulate host immunity, with particular focus on their ability to suppress IFN-I signalling, reprogramming of cellular metabolism, and activate inflammatory and apoptotic pathways. We further examine how mutations, especially those arising in the Omicron variant, have reshaped APs structure and function during viral evolution.

SARS-CoV-2 APs effectively suppress the IFN-I response, a critical antiviral pathway that restricts viral replication. The widespread targeting of multiple steps within this signalling cascade by several APs highlights the essential role of IFN-I inhibition in facilitating efficient viral infection. Patients with severe COVID-19 often exhibit an exaggerated inflammatory response characterized by elevated levels of proinflammatory cytokines such as IL-6, IL-1β, IL-11, TNF-α, IL-8, and IL-10. This dysregulated “cytokine storm” contributes to tissue damage, multiorgan dysfunction, and in some cases, fatal outcomes. While many SARS-CoV-2 APs appear to promote this hyperinflammatory state, others may exert mitigating effects. Future studies should dissect the temporal dynamics between IFN-I pathway suppression and the subsequent induction of the proinflammatory response. One plausible hypothesis is that viral proteins initially suppress both antiviral and inflammatory signalling to favour replication, followed by selective activation of inflammation to facilitate immune evasion. In this context, dysregulated inflammation may paradoxically benefit SARS-CoV-2 by causing collateral tissue damage, recruiting new susceptible cells and generating an “immune chaos” that impairs coordinated antiviral responses, ultimately leading to immune T-cell exhaustion and impaired viral clearance (134).

SARS-CoV-2 APs modulate host apoptotic pathways via multiple mechanisms, enabling the virus to either induce apoptosis in immune cells to evade host defences or delay apoptosis in infected cells to prolong viral replication. This mechanistic insight reveals potential therapeutic opportunities, including targeting caspase activation, modulating potassium channels, or regulating endoplasmic reticulum (ER) stress responses to reduce virus-induced tissue damage. However, the overlapping and interconnected nature of apoptosis, pyroptosis, and ferroptosis complicates selective therapeutic targeting of these pathways. Future research should validate these mechanisms in animal models and patient-derived systems, while exploring how host genetic factors influence susceptibility to virus-induced cell death. Such knowledge is critical for developing targeted therapies against COVID-19 and related viral infections.

Metabolic effects of SARS-CoV-2 APs are likely context-dependent, varying according to cell type and host factors. These metabolic alterations are especially relevant in Long COVID, a major health concern characterized by prolonged symptoms such as fatigue, neurocognitive dysfunction, and chronic inflammation. Persistent mitochondrial dysfunction, unresolve inflammation, and impaired autophagy may underlie some of these symptoms. Several APs, including ORF6 and ORF8, can inhibit IFN-I signalling and MHC-I surface expression, respectively, potentially delaying viral clearance and promoting chronic immune activation (40, 135). Likewise, ORF7a and ORF3a induce inflammatory cytokines (IL-1β, IL-6 and TNF-α), which are elevated in both severe COVID-19 and Long COVID (79, 136–138). These proteins also promote apoptosis and disrupt tight junctions, potentially contributing to the microvascular injury observed in PASC (96, 98, 139). Notably, APs may persist within protected intracellular niches, including mitochondria and the endoplasmic reticulum, allowing them to continue disrupting host cell metabolism and stress response even after viral clearance (101). This persistent activity could drive chronic mitochondrial dysfunction, oxidative stress, and low-grade inflammation, consistent with Long COVID pathology (140). These findings position viral APs as prime suspects in the pathogenesis of Long COVID and highlight the need for longitudinal, patient-derived studies that directly assess APs persistence, subcellular localization and metabolic impact, integrating single-cell multi-omics and functional assays. Such approaches could uncover AP-specific biomarkers of disease persistence and identify therapeutic targets for early intervention.

The relationship between the evolution of SARS-CoV-2 APs and host responses remains largely unexplored. Since the emergence of Omicron, numerous mutations have become globally fixed; yet, their effects on IFN-I signalling, inflammation, apoptosis, and metabolic reprogramming are still unclear. Notably, Omicron is phylogenetically more distant from the original Wuhan strain than any previously characterized variant (141). This suggests a fundamental shift in virus-host interactions and, consequently, a potentially altered role for APs during infection—similar to what has been observed with the spike protein (142).

Despite these advances, significant challenges remain. Most studies to date have been conducted *in vitro*, often relying on overexpression of individual APs without accounting for potential synergistic effects among APs or interactions with other viral components. This review highlights how individual APs modulate IFN-I signalling, inflammatory responses, apoptosis, and metabolic reprogramming. However, the coordinated expression of multiple APs may exert synergistic or compensatory effects that cannot be captured in isolated overexpression systems. Furthermore, several non-structural SARS-CoV-2 proteins encoded within *ORF1ab* also influence viral pathogenicity (2, 143), further complicating functional interpretation. To overcome these limitations, methodological approaches should evolve to better dissect the mechanisms of SARS-CoV-2 infection. Reverse genetics strategies, such as CRISPR-mediated deletion or combinatorial knockouts of multiple APs, could clarify their individual and collective contributions, as demonstrated for some SARS-CoV, SARS-CoV-2 and MERS-CoV APs (73, 144, 145). Complementary use of patient-derived organoids, which preserve tissue-specific architecture and cellular diversity, would enable more physiological relevant modelling of virus-host interactions. Integration of spatial proteomics to map protein localization and interaction networks *in situ* together with single-cell transcriptomics of biopsy or autopsy tissues, could further uncover cell type-specific responses and synergistic APs effects during infection. within physiologically relevant contexts.

Additionally, *in vivo* models are urgently needed to confirm *in vitro* findings and to guide development of therapeutics targeting key signalling components to reduce COVID-19 severity. Future research should incorporate Omicron-derived APs variants into both *in vitro* and *in vivo* experimental systems. Moreover, integrating computational modelling, structural biology and bioinformatics with animal and clinical data from Omicron-infected individuals will be essential to fully elucidate the evolving dynamics of SARS-CoV-2 APs in host modulation.
