# Supplementary material for: Hidden players of COVID-19: the evolving roles of SARS-CoV-2 accessory proteins
Source: Front Immunol. 2025 Nov 28;16:1726698. doi: 10.3389/fimmu.2025.1726698 (PMC12698434; doi:10.3389/fimmu.2025.1726698)
Supplement: Supplementary file 1 [file Table1.docx]

**Supplementary Table 1. General information on SARS-CoV-2 APs.** APs are classified in two groups: Infection-related and Putative APs Additionally, their genomic coordinates, number of amino acid residues, active conformation state and subcellular localization are included. *NR: Not reported; ER: Endoplasmic reticulum; PM: plasma membrane; ERGIC: Endoplasmic reticulum Golgi intermediate compartment

| **Accessory protein group** | **ORF** | **Genome position** | **Number of amino acid residues** | **Active conformation^*^** | **Subcellular localization^*^** | **References** |
| --- | --- | --- | --- | --- | --- | --- |
| Infection-related | 2b | 21,744 - 21,863 | 39 aa | NR | NR | (1,2) |
|  | 3a | 25,393 - 26,220 | 275 aa | Dimer  Tetramer | Cytoplasm  Endosomes  ER  Golgi  PM  Lysosomes | (3–6) |
|  | 3c | 25,457 – 25,582 | 41 aa | Monomer | Cytoplasm  Mitochondria | (7,8) |
|  | 3d-2 | 25,596 – 25,697 | 33 aa | NR | NR | (2,7) |
|  | 6 | 27,202 – 27,387 | 61 aa | Monomer | Cytoplasm  ER  Golgi | (3,9) |
|  | 7a | 27,394 – 27,759 | 121 aa | Monomer | Cytoplasm  ER  ERGIC  Golgi  Perinuclear region | (3,10–13) |
|  | 7b | 27,756 – 27,887 | 43 aa | Tetramer | Cytoplasm  Endosomes  ER  Golgi  PM  Mitochondria | (3,13,14) |
|  | 8 | 27,894 – 28,259 | 121 aa | Dimer | Cytoplasm  ER  Golgi  Extracellular  Nucleus | (3,15–18) |
|  | 9b | 28,284 – 28,577 | 97 aa | Dimer  Monomer | Cytoplasm  Mitochondria | (3,19–21) |
| Putative | 3b | 25,814 – 25,882 | 22 aa | NR | Cytosol | (22) |
|  | 3d | 25,524 – 25,697 | 57 aa | NR | NR | (2,7) |
|  | 9c | 28,734 – 28,955 | 73 aa | NR | Cytoplasm | (3) |
|  | 10 | 29,558 – 29,674 | 38 aa | Monomer | ER  Mitochondria | (3,23,24) |

**References**

1. Weingarten-Gabbay S, Klaeger S, Sarkizova S, Pearlman LR, Chen DY, Gallagher KME, Bauer MR, Taylor HB, Dunn WA, Tarr C, et al. Profiling SARS-CoV-2 HLA-I peptidome reveals T cell epitopes from out-of-frame ORFs. *Cell* (2021) 184:3962-3980.e17. doi: 10.1016/j.cell.2021.05.046

2. Finkel Y, Mizrahi O, Nachshon A, Weingarten-Gabbay S, Morgenstern D, Yahalom-Ronen Y, Tamir H, Achdout H, Stein D, Israeli O, et al. The coding capacity of SARS-CoV-2. *Nature* (2021) 589:125–130. doi: 10.1038/s41586-020-2739-1

3. Gordon DE, Hiatt J, Bouhaddou M, Rezelj V V., Ulferts S, Braberg H, Jureka AS, Obernier K, Guo JZ, Batra J, et al. Comparative host-coronavirus protein interaction networks reveal pan-viral disease mechanisms. *Science (1979)* (2020) 370: doi: 10.1126/science.abe9403

4. Kern DM, Sorum B, Mali SS, Hoel CM, Sridharan S, Remis JP, Toso DB, Kotecha A, Bautista DM, Brohawn SG. Cryo-EM structure of SARS-CoV-2 ORF3a in lipid nanodiscs. *Nat Struct Mol Biol* (2021) 28:573–582. doi: 10.1038/s41594-021-00619-0

5. Zhang X, Yang Z, Pan T, Long X, Sun Q, Wang PH, Li X, Kuang E. SARS-CoV-2 ORF3a induces RETREG1/FAM134B-dependent reticulophagy and triggers sequential ER stress and inflammatory responses during SARS-CoV-2 infection. *Autophagy* (2022) 18:2576–2592. doi: 10.1080/15548627.2022.2039992

6. Miao G, Zhao H, Li Y, Ji M, Chen Y, Shi Y, Bi Y, Wang P, Zhang H. ORF3a of the COVID-19 virus SARS-CoV-2 blocks HOPS complex-mediated assembly of the SNARE complex required for autolysosome formation. *Dev Cell* (2021) 56:427-442.e5. doi: 10.1016/j.devcel.2020.12.010

7. Müller M, Herrmann A, Fujita S, Uriu K, Kruth C, Strange A, Kolberg JE, Schneider M, Ito J, Müller MA, et al. ORF3c is expressed in SARS‐CoV‐2‐infected cells and inhibits innate sensing by targeting MAVS. *EMBO Rep* (2023) 24: doi: 10.15252/embr.202357137

8. Stewart H, Lu Y, O’Keefe S, Valpadashi A, Cruz-Zaragoza LD, Michel HA, Nguyen SK, Carnell GW, Lukhovitskaya N, Milligan R, et al. The SARS-CoV-2 protein ORF3c is a mitochondrial modulator of innate immunity. *iScience* (2023) 26: doi: 10.1016/j.isci.2023.108080

9. Wong HT, Cheung V, Salamango DJ. Decoupling SARS-CoV-2 ORF6 localization and interferon antagonism. *J Cell Sci* (2022) 135: doi: 10.1242/jcs.259666/274474

10. Zhou Z, Huang C, Zhou Z, Huang Z, Su L, Kang S, Chen X, Chen Q, He S, Rong X, et al. Structural insight reveals SARS-CoV-2 ORF7a as an immunomodulating factor for human CD14+ monocytes. *iScience* (2021) 24: doi: 10.1016/j.isci.2021.102187

11. Martin-Sancho L, Lewinski MK, Pache L, Stoneham CA, Yin X, Becker ME, Pratt D, Churas C, Rosenthal SB, Liu S, et al. Functional landscape of SARS-CoV-2 cellular restriction. *Mol Cell* (2021) 81:2656-2668.e8. doi: 10.1016/j.molcel.2021.04.008

12. Arshad N, Laurent-Rolle M, Ahmed WS, Hsu JCC, Mitchell SM, Pawlak J, Sengupta D, Biswas KH, Cresswell P. SARS-CoV-2 accessory proteins ORF7a and ORF3a use distinct mechanisms to down-regulate MHC-I surface expression. *Proc Natl Acad Sci U S A* (2023) 120: doi: 10.1073/pnas.2208525120

13. García-García T, Fernández-Rodríguez R, Redondo N, de Lucas-Rius A, Zaldívar-López S, López-Ayllón BD, Suárez-Cárdenas JM, Jiménez-Marín Á, Montoya M, Garrido JJ. Impairment of antiviral immune response and disruption of cellular functions by SARS-CoV-2 ORF7a and ORF7b. *iScience* (2022) 25: doi: 10.1016/j.isci.2022.105444

14. Xiao X, Fu Y, You W, Huang C, Zeng F, Gu X, Sun X, Li J, Zhang Q, Du W, et al. Inhibition of the RLR signaling pathway by SARS-CoV-2 ORF7b is mediated by MAVS and abrogated by ORF7b-homologous interfering peptide. *J Virol* (2024) 98: doi: 10.1128/jvi.01573-23

15. Flower TG, Buffalo CZ, Hooy RM, Allaire M, Ren X, Hurley JH. Structure of SARS-cov-2 ORF8, a rapidly evolving immune evasion protein. *Proc Natl Acad Sci U S A* (2021) 118:e2021785118. doi: 10.1073/pnas.2021785118

16. Matsuoka K, Imahashi N, Ohno M, Ode H, Nakata Y, Kubota M, Sugimoto A, Imahashi M, Yokomaku Y, Iwatani Y. SARS-CoV-2 accessory protein ORF8 is secreted extracellularly as a glycoprotein homodimer. *Journal of Biological Chemistry* (2022) 298: doi: 10.1016/j.jbc.2022.101724

17. Wu X, Xia T, Shin WJ, Yu KM, Jung W, Herrmann A, Foo SS, Chen W, Zhang P, Lee JS, et al. Viral Mimicry of Interleukin-17A by SARS-CoV-2 ORF8. *mBio* (2022) 13: doi: 10.1128/mbio.00402-22

18. Chen J, Lu Z, Yang X, Zhou Y, Gao J, Zhang S, Huang S, Cai J, Yu J, Zhao W, et al. Severe Acute Respiratory Syndrome Coronavirus 2 ORF8 Protein Inhibits Type I Interferon Production by Targeting HSP90B1 Signaling. *Front Cell Infect Microbiol* (2022) 12: doi: 10.3389/fcimb.2022.899546

19. Jiang H wei, Zhang H nan, Meng Q feng, Xie J, Li Y, Chen H, Zheng Y xiao, Wang X ning, Qi H, Zhang J, et al. SARS-CoV-2 Orf9b suppresses type I interferon responses by targeting TOM70. *Cell Mol Immunol* (2020) 17:998–1000. doi: 10.1038/s41423-020-0514-8

20. Brandherm L, Kobaš AM, Klöhn M, Brüggemann Y, Pfaender S, Rassow J, Kreimendahl S. Phosphorylation of sars-cov-2 orf9b regulates its targeting to two binding sites in tom70 and recruitment of hsp90. *Int J Mol Sci* (2021) 22: doi: 10.3390/ijms22179233

21. Gao X, Zhu K, Qin B, Olieric V, Wang M, Cui S. Crystal structure of SARS-CoV-2 Orf9b in complex with human TOM70 suggests unusual virus-host interactions. *Nat Commun* (2021) 12: doi: 10.1038/s41467-021-23118-8

22. Konno Y, Kimura I, Uriu K, Fukushi M, Irie T, Koyanagi Y, Sauter D, Gifford RJ, Nakagawa S, Sato K. SARS-CoV-2 ORF3b Is a Potent Interferon Antagonist Whose Activity Is Increased by a Naturally Occurring Elongation Variant. *Cell Rep* (2020) 32: doi: 10.1016/j.celrep.2020.108185

23. Li X, Hou P, Ma W, Wang X, Wang H, Yu Z, Chang H, Wang T, Jin S, Wang X, et al. SARS-CoV-2 ORF10 suppresses the antiviral innate immune response by degrading MAVS through mitophagy. *Cell Mol Immunol* (2022) 19:67–78. doi: 10.1038/s41423-021-00807-4

24. Zhu K, Song L, Wang L, Hua L, Luo Z, Wang T, Qin B, Yuan S, Gao X, Mi W, et al. SARS-CoV-2 ORF10 hijacking ubiquitination machinery reveals potential unique drug targeting sites. *Acta Pharm Sin B* (2024) 14:4164–4173. doi: 10.1016/j.apsb.2024.05.018
